# Is Gauchian genotyping of *GBA1* variants reliable?

**DOI:** 10.1038/s42003-025-08059-y

**Published:** 2025-05-09

**Authors:** Nahid Tayebi, Jens Lichtenberg, Ellen Hertz, Ellen Sidransky

**Affiliations:** 1 Medical Genetics Branch, National Human Genome Research Institute, National Institutes of Health, Bethesda, MD USA; 2 Aligning Science Across Parkinson’s (ASAP) Collaborative Research Network, Chevy Chase, MD USA

**Keywords:** Neuroscience, Genetics, Neurology

**arising from**: M. Toffoli et al. *Communications Biology* 10.1038/s42003-022-03610-7 (2022)

Gaucher disease (GD) results from biallelic pathogenic variants in the gene *GBA1*, which encodes the lysosomal enzyme glucocerebrosidase (E.C.3.2.1.45). Over 500 pathogenic variants in *GBA1*, located on chromosome 1q21, have been identified in patients with GD^1,2^. Variants in *GBA1* are also the most common known genetic risk factor for Parkinson disease (PD) and dementia with Lewy bodies (DLB)^3–6^. The presence of a highly homologous *GBA1* pseudogene, *GBAP1*, located approximately 16 kb downstream from the gene^7^, complicates variant detection and sequence analyses, as highly homologous pseudogenes increase the frequency of nonequal pairing of chromosomes, resulting in complex recombinant alleles^8–10^ (Fig. 1). While *GBAP1* is 96% homologous to *GBA1* in exonic regions, this sequence similarity increases to ~98% between intron 8 to the 3’untranslated region (UTR), where a 55 bp deletion in exon 9 is the major exonic difference^7,11^. Contiguous to *GBA1* and *GBAP1* are the gene *MTX1* and its pseudogene *MTX1P*, which also tend to generate DNA rearrangements^12,13^ (Fig. 1). Some Gaucher-related variants are present in *GBAP1*^11,14^. Furthermore, gene-pseudogene DNA rearrangements comprise a significant proportion of mutant *GBA1* alleles, and such alterations have been detected at different sites between intron 2 to the 3’UTR^1,15,16^. Over twenty different recombinant alleles have been described^17–19^, with Rec*NciI*, RecTL, and RecTL+55 bp being the most common. Direct Sanger sequencing, quantitative real-time PCR, Southern blotting, and lately, WGS have been utilized to detect the most common recombinant alleles^1,17,20^ both for the diagnosis of GD and PD research^10,21–23^.

**Fig. 1:**
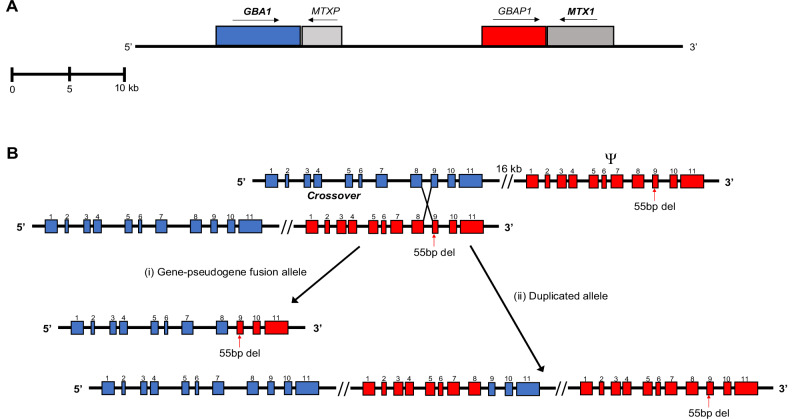
Two nearby genes, *GBA1* and *MTX1* and their pseudogenes. **A** Illustration of a portion of chromosome 1q21 demonstrating the physical relationship between the genes *GBA1* and *MTX1*, and their homologous pseudogenes with sense and antisense orientation shown. **B** Schematic presentation of a reciprocal cross-over between homologous regions resulting in two possible gene rearrangements: (i) a fusion between the gene and its pseudogene resulting in a deletion of the intergenic region, and (ii) a recombination resulting in a third sequence containing a partial duplication of the pseudogene and several duplicated exons from the gene sequence. The two slanted vertical lines indicate regions omitted in the diagram. The X indicates the site of recombination. del deletion, bp base pairs.

Recently, Toffoli et al. introduced the software tool Gauchian^24^ to establish *GBA1* variants, including point mutations and recombinant alleles from short-read whole genome sequencing (WGS) data. Using the sequencing read depth across the 10 kb intergenic region between *GBA1* and *GBAP1* as a landmark, Gauchian employs a Gaussian mixture model to call copy number variants, with losses of the intergenic region presumed to represent pathogenic fusion alleles consisting of partial *GBA1* and *GBAP1* fragments, and gains corresponding to an allele with duplication of the 10 kb intergenic fragment. The size of deleted (copy number loss) or duplicated (copy number gain) fragments depends on the cross-over site between *GBA1* and *GBAP1* and is not considered by Gauchian, which therefore may fail to detect some recombinant alleles. Gauchian utilizes 82 sites that differ between *GBA1* and *GBAP1* to identify the breakpoints between gene and pseudogene variants and uses the 1.1 kb homology region in exons 9 to 11, which includes 10 SNPs and a 55 bp deletion in exon 9 of *GBAP1*, to uniquely identify whether a fragment is derived from the gene or pseudogene. This strategy can help to characterize *GBA1*/*GBAP1* fusion genes, gene conversions, and duplicated alleles in the region, so long as the cross-over occurs in this 1.1 kb region. Due to its ease of use, low-performance requirements, and straight-forward output, Gauchian has already been integrated into automatic pipelines, such as Illumina’s Dragen4, and is incorporated into WGS workflows for neurodegenerative diseases, as it enables quick and easy *in-silico* identification of *GBA1* variants.

We compared calls made by Gauchian in 95 samples (three alleles carried two variants in *cis*) from our GD cohort to genotypes established via Sanger sequencing (Supplementary Data 1). Gauchian predicted the *GBA1* genotype correctly in 84 samples (Table 1). In three of the eleven discrepant results, Gauchian was unable to establish a fragment copy number count and reported “None”. Given that our sequencing depth was high-coverage (depth > 30X), it is not clear why the software still eliminates some samples from the analysis. When one of the three cases was aligned to hg38, the program no longer reported “None”, demonstrating that the sequencing depth was indeed adequate. Genotype visualization generated from WGS and Sanger data for each case misidentified by Gauchian (Supplementary Figs. 1, A–J) clearly shows the omitted variant calls for the three “no call” samples. While a case can be made that these samples should be disregarded as false negatives, it is still worth noting that even a visual inspection of the data easily confirmed the genotype.

**Table 1:** Eleven cases where the Gauchian genotype predictions were not validated by Sanger sequencing

	Gauchian predictions				Sanger assessment	
Sample	Biallelic/ Carrier	Copy number of *GBA1* and *GBAP1*	*GBAP1*-like variants in exon 9-11	“Unphased” (*GBA1*-specific) variants	Genotype	Prediction
Pat_03	False/ False	4		p.Asn409Ser	p.Asn409Ser/ p.Asn409Ser	False Negative
Pat_08	False/ False	4		p.Asn409Ser	p.Asn409Ser/ p.Gln389Ter	False Negative
Pat_16	False/ Carrier	3	c.1263del+RecTL	p.Asn409Ser p.Asn409Ser	p.Asn409Ser c.1263del+RecTL	False Positive
Pat_26	False/ False	4		p.Asn409Ser	p.Asn409Ser/ p.Arg502His	False Negative
Pat_28	False/ False	4		p.Arg535His	p.Arg535His/ p.Cys381Tyr	False Negative
Pat_47	False/ False	4		p.Asn409Ser	p.Asn409Ser/ p.Leu483Pro	False Negative
Pat_58	False/ False	4		p.Asn409Ser/ p.Arg296Ter	*p.Asn409Ser p.Arg296Ter c.203delC	False Negative
Pat_92	Biallelic/ False	7	p.Asp448His/ p.Leu483Pro, p.Asp448His		p.Asp448His/ p.Leu483Pro+Rec7	False Negative
Pat_75	None/ None	None			p.Arg502Cys/ p.Arg159Trp	Missed
Pat_76	None/ None	None			p.Asn409Ser/ p.Asn409Ser	Missed
Pat_79	None/ None	None			p.Leu483Pro/ p.Arg502Cys	Missed

The first four columns represent the output from Gauchian, and the last two columns show the Sanger-established genotype and the prediction assessment for correctness. An asterisk denotes Sanger-established alleles that could not be assigned a phase.

Examining the performance of Gauchian based on the individual alleles (Table 2 and Supplementary Data 2), of the 193 missense mutations and recombinant alleles, 26 alleles were incorrectly identified, corresponding to an error rate of 13.40%. Of particular concern were eleven alleles erroneously predicted as wildtype. Furthermore, there were instances of failure to detect variants p.Asn409Ser (N370S) and p.Leu483Pro (L444P), which are the most common Gaucher mutations and represent crucial landmarks for clinical genotyping. Some rare *GBA1* mutations, e.g., p.Cys381Tyr (C342Y), p.Gln389Ter (Q350X), and p.Gly234Glu (G195E), and single nucleotide deletions (frameshift mutations) such as c.203del, were not called as they were not annotated in Gauchian’s internal database of known variants. The inability to detect one p.Arg502His (R463H) and three p.Arg502Cys (R463C) alleles out of a total of six cases in the cohort is concerning, since they are present in the ClinVar database. Across all alleles characterized by Sanger sequencing, Gauchian achieved a sensitivity of 0.9275 and specificity of 0.9977 (Supplementary Data 3). When excluding the six alleles that Gauchian eliminated due to uneven coverage, the sensitivity increased to 0.9572 with the same specificity.

**Table 2:** Gauchian performance evaluating individual alleles

	Benchmark (Sanger)		Gauchian (b37)				Gauchian (hg38)			
Variant	POS	NEG	TP	TN	FP	FN	TP	TN	FP	FN
p.Asn409Ser	122	71	119	70	1	3	114	71	0	8
Rec7	1	192	0	192	0	1	0	192	0	1
c.1263del	3	190	3	190	0	0	3	190	0	0
p.Leu29Alafs*18	8	185	8	185	0	0	8	185	0	0
c.203del	1	192	0	192	0	1	0	192	0	1
p.Cys381Tyr	1	192	0	192	0	1	0	192	0	1
p.Asp448His	2	191	2	191	0	0	2	191	0	0
p.Phe252Ile	1	192	1	192	0	0	1	192	0	0
p.Gly234Glu	1	192	1	192	0	0	1	192	0	0
p.Gly241Arg	1	192	1	192	0	0	1	192	0	0
p.Gly416Ser	1	192	1	192	0	0	1	192	0	0
c.115+1 G > A	3	190	3	190	0	0	3	190	0	0
p.Leu483Pro	20	173	17	173	0	3	17	173	0	3
p.Gln389Ter	1	192	0	192	0	1	0	192	0	1
p.Arg159Trp	2	191	1	191	0	1	1	191	0	1
p.Arg209Cys	1	192	1	192	0	0	1	192	0	0
p.Arg296Gln	1	192	1	192	0	0	1	192	0	0
p.Arg296Gln	1	192	1	192	0	0	1	192	0	0
p.Arg324Cys	1	192	1	192	0	0	1	192	0	0
p.Arg398Ter	1	192	1	192	0	0	1	192	0	0
p.Arg502Cys	6	187	3	187	0	3	4	187	0	2
p.Arg535His	2	191	2	191	0	0	2	191	0	0
c.1263del+RecTL	1	192	1	192	0	0	1	192	0	0
Rec*NciI*	2	191	2	191	0	0	2	191	0	0
p.Val391Leu	1	192	1	192	0	0	1	192	0	0
p.Val433Leu	2	191	2	191	0	0	2	191	0	0
p.Arg296Gln	1	192	1	192	0	0	1	192	0	0
WT	5	188	5	177	11	0	6	171	16	0

True positives (TP) and true negatives (TN) refer to the number of correctly identified allele calls (POS) and absences of calls (NEG) for a specific variant, while false positives (FP) represent Gauchian’s predictions that have been validated as absent (NEG). False negatives (FN) are omissions validated as observed variants (POS).

The inability of Gauchian to detect the most common *GBA1* variants on both alleles in three patients (Pats_75, 76 and 79) with confirmed GD was also of concern. As can be visualized in the screenshots of Sanger sequence shown in Supplementary Fig. 1G-I (Pats_75,76,79), our coverage at the site of the mutated nucleotide is very clear.

Since Gauchian supports the analysis of sequencing data aligned to different references of the human genome, and previous work by Pan et al. has shown significant differences between single nucleotide variants using different reference genomes^25^, we compared the performance of the software with both the b37 (hg19) and hg38 versions (Supplementary Data 4). While most predictions were congruent, there were four cases with distinct differences. In Pat_16, Sanger sequencing showed heterozygosity for p.Asn409Ser. When b37 was used, Gauchian predicted a homozygous p.Asn409Ser genotype, and with hg38, no mutation was identified. For Pats_35 and 78, Gauchian correctly identified a homozygous p.Asn409Ser genotype using b37 as a reference, but missed both mutant alleles entirely when using hg38. For Pat_75, Gauchian missed both Sanger-identified variants (p.Arg502Cys and p.Arg159Trp) when using b37 but reported the p.Arg502Cys allele correctly with hg38. There were further discrepancies between the copy numbers reported: for Pats_35 and 75, hg38 reported CN = 3, while b37 predicted CN = 4 in Pat_35 and made no call for Pat_75. The issue of miscalls related to reference alignment was raised previously unrelated to Gauchian in a study of *GBA1* variants in Multiple System Atrophy^26^.

Among the 95 cases evaluated, four carried recombinant alleles, two with Rec*NciI*, one with RecTL+ c.1263del and one with a Rec7 (a rare duplication that includes the *GBA1* gene and duplicated pseudogene)^17^ (Table 3). Gauchian reported both Rec*NciI* cases correctly (Table 3); however, a detailed analysis of the Binary Alignment Map (BAM) file generated during the alignment of the WGS data for one of them (Pat_95) did not identify the known Rec*NciI* mismatches in exon 10 (p.Leu483Pro, p.Ala495Pro, and p.Val499Val), but identified 3’UTR mismatches associated with *GBAP1* (Supplementary Fig. 2). The intron 9 and the 3’-UTR *GBAP1* mismatches indicate a fusion allele (a cross-over between *GBA1* and its pseudogene resulting in the absence of one copy of *GBA1P* and the intergenic region). Therefore, only three copies of the gene and pseudogene should be reported, rather than the four copies identified by Gauchian. For the other Rec*NciI* case (Pat_71), only p.Leu484Pro and not the other two mismatches in exon 10 or 3’UTR *GBA1*/*GBAP1* mismatches could be visually identified by WGS (Supplementary Fig. 2). For Pat_16 with Rec*TL* + c.1263del (Table 3) the WGS BAM file failed to show the expected exon 9-11 gene/pseudogene mismatches (p.Asp448His, p.Leu483Pro, p.Ala495Pro, and p.Val499Val) (Supplementary Fig. 2). Here, Gauchian correctly reported the copy number to be three for this allele, resulting from a cross-over between the gene and pseudogene in intron 8. This generated a fusion-gene lacking the intergenic region and one copy of the pseudogene (Fig. 1). However, Gauchian was not able to detect the recombinant mutation on the second allele and misreported the heterozygous p.Asn409Ser mutation as homozygous.

**Table 3:** Patients with recombinant alleles and abnormal *GBA1* + *GBAP1* copy numbers

	Gauchian prediction					Sanger assessment
Sample	Biallelic/ Carrier	Copy Number of *GBA1* and *GBAP1*	*GBA1* Deletion Breakpoint	*GBAP1*-like Variants in Exons 9-11	“Unphased” (*GBA1*-specific) Variants	Genotype
Pat_16	False/ Carrier	3	True	c.1263del+RecTL	p.Asn409Ser p.Asn409Ser	p.Asn409Ser c.1263del+RecTL
Pat_71	False/ Carrier	4		Rec*NciI*		Rec*NciI* WT
Pat_95	False/ Carrier	4		Rec*NciI*	p.Asn409Ser	p.Asn409Ser Rec*NciI*
Pat_42	False/ False	6			p.Arg398Ter p.Val391Leu	p.Arg398Ter p.Val391Leu
Pat_72	False/ False	5			p.Gly241Arg	p.Gly241Arg WT
Pat_92	Biallelic/ False	7		p.Asp448His p.Leu483Pro, p.Asp448His		p.Asp448His p.Leu483Pro+Rec7

The first five columns represent the output from Gauchian, and the last column shows the Sanger-established genotype.

In some cases, Gauchian misreported copy number gains, as seen with the third type of recombinant allele, Rec7, resulting in a *GBAP1* duplication (Pat_92, Table 3). This rare recombination event is the most difficult to identify^10,17,27^. The site of cross-over is between *GBAP1* and *GBA1* or between the contiguous gene *MTX1* and *MTX1P*, causing a complete *GBAP1* duplication (Supplemental figure 2)^10^. Careful evaluation of the WGS from Pat_92 demonstrated changes in the read depth of the intergenic area compared to the rest of the genome (Supplementary Fig. 2). Instead of one extra copy of the pseudogene, Gauchian indicated three extra copies, reporting a copy number of seven. In two additional cases, Gauchian identified increased copy numbers (Pats_42 and 72, Table 3) without signs of a recombinant allele, meriting further investigation.

Another genetic event resulting in variants detected in this cohort is gene conversion. Four patients carried an allele with c.1263del (55 bp del) in exon 9. While Gauchian called the deletion correctly, it identified a gene fusion event (represented as a copy number loss) only in one of the cases (RecTL+55 bp in Pat_16).

To further evaluate whether Gauchian may falsely report *GBA1* variants, both low and high-coverage genomes from phase 3 of the 1000 Genomes project^28^ were extracted and run using the software. Gauchian called biallelic variants in 20 cases (28 variants total) for the low coverage genomes, all called as a *GBAP1*-like variant in exons 9-11, and eight other unphased (non-pseudogene) variants. However, no biallelic calls were made when Gauchian was run on the higher coverage 1000 genomes (Supplementary Data 5). Among the 2706 cases with high coverage, Gauchian did detect 12 *GBA1* variant carriers, all with a *GBAP1*-like variant in exon 9–11, and 45 cases with other *GBA1* variants, in addition to five “no calls”. Of interest is the case NA18997, which is reported to have two variants but is not called as biallelic. The most frequent variants detected by Gauchian were two variants p.Glu365Lys (E326K) and p.Thr408Met (T369M), which are relatively common in the general population. Considering both our Gaucher cohort and the 1000 Genomes high coverage cohort, Gauchian achieved a sensitivity of 0.9275, specificity of 0.9985, precision of 0.9372, and accuracy of 0.9968 (Supplementary Data 6). We further recommend that the tool provide an input filter that prevents low coverage or unsuitable data from being processed by Gauchian and reported as “no-calls.”

Even when Gauchian accurately identified recombinant alleles, it could not demonstrate the underlying recombination mechanism. For example, Rec*NciI* may result from gene fusion or conversion. WGS alone also could not accurately delineate many of the recombinant alleles, and thus a combination of approaches is needed to detect and define recombinant *GBA1* alleles accurately. Perhaps the use of long-read WGS will ultimately be helpful.

We found that with Sanger-established Rec alleles, when 60 or more nucleotides of pseudogene sequence were present, detecting the Rec alleles visually based on short-read sequencing and comparing mismatches between gene and pseudogene was particularly challenging. Gauchian performed remarkedly well in this situation and recognized the Rec allele. However, in these cases, we observed that a missense variant in trans was sometimes erroneously reported by Gauchian as a homozygous mutation, as seen with patients 16 and 92 (Tables 1, 3). The problem appears to be that with the short reads, a gene-pseudogene rearrangement fragment downstream of *GBA1* erroneously aligns with the reference pseudogene sequence. When Rec alleles resulted from a gene-conversion event (incorporating a short sequence of *GBA1P*), Gauchian detected the variants correctly, as shown with Pats_71 and 95 (Table 3).

In conclusion, the tool currently has several limitations. It is unable to identify even some relatively common variants and other rare or de novo mutations, as currently it can only detect the 121 known *GBA1* variants and three recombinant alleles included in its database. The database does not appear to be updated regularly. Due to this limited number of variants, the software may incorrectly report a carrier or even a patient with GD as normal, which can have clinical repercussions and distort PD data analyses. With the broader use of next-generation sequencing, more variants are continually being identified that should be added to Gauchian. Also, while existing benchmarking approaches have favored the newer hg38^26^, Gauchian performs better with the older reference version (b37) for most common genotypes. We recommend adding an input filter to Gauchian to prevent unsuitable data from being processed. Furthermore, the authors should consider using additional sources other than ClinVar for their variant list, such as GnomAD (Genome Aggregation Database) which includes large-scale human genomic data from different populations, the HGMD (Human Gene Mutation Database) and/or other *GBA1*-specific databases. Since the tool has challenges in identifying copy numbers and structural variations, the Population Genomics and Structural Variants, and DECIPHER databases would also be helpful. We emphasize that any results used for clinical purposes be validated with Sanger sequencing or by using at least another method to evaluate the WGS. This is especially critical when using Gauchian to genotype newborns or children suspected of having Gaucher disease. Future studies should specifically compare the results of Sanger sequencing, copy number analyses, and WGS with Gauchian predictions in larger cohorts, and include additional individuals with known complex alleles. While Gauchian may be useful when evaluating large cohorts for research purposes, it should not be used as a stand-alone black box test in diagnostic variant-calling pipelines like Dragen4 for high-throughput genotyping, or as the basis for patient counseling.

## Methods

High molecular weight DNA was extracted from samples from 95 patients (90 with GD and five known carriers of *GBA1* variants) seen under an NIH Institutional Review Board-approved natural history study with informed consent. Each sample underwent both Sanger sequencing and WGS, performed at the NIH Intramural Sequencing Center.

*GBA1-*related genotypes for each patient were established using Sanger sequencing with primers and protocols introduced by Stone et al.^29^ WGS sequencing libraries were generated from 1 μg genomic DNA using a TruSeq® DNA PCR-Free HT Sample Preparation Kit (Illumina, #FC-121-3001), with median insert sizes of approximately 400 bp. Libraries were tagged with barcodes to allow pooling and were sequenced on the NovaSeq 6000 (Illumina, RRID:SCR_016387) to obtain at least 300 million 151-base read pairs per individual library. The bioinformatics pipeline followed guidelines for the Genome Analysis Toolkit (GATK, RRID:SCR_001876)^30^. Unless otherwise specified, reads were aligned to the human b37_decoy reference sequence (UCSC assembly hg19, NCBI build 37) using BWA (RRID:SCR_010910, http://bio-bwa.sourceforge.net/ accessed on 22 September 2022)^31^. Gauchian (v.1.0.2, https://github.com/Illumina/Gauchian accessed on 18 March 2022)^24^ was run using the b37 reference genome.

To evaluate the performance of Gauchian against the assembled cohort of patients with GD, we used Sanger calls for a specific known mutation as positive observations across the afflicted alleles and the absence of the same mutation in an allele as negative observations. For example, if a patient was observed to have genotype p.Asn409Ser/p.Asn409Ser, the p.Asn409Ser variant would be assigned two positive observations and 0 negative ones, while for this patient, a p.Leu483Pro variant would be assigned 0 positive observations and two negative ones. After mapping all the variants observed across the cohort, it was then possible to categorize each Gauchian prediction (or absence of prediction) into (1) a true positive call, where Gauchian detected the correct Sanger variant, (2) a true negative call, where Gauchian correctly omitted a variant, (3) a false positive call, where Gauchian predicted a variant in the patient, but Sanger and visual inspection of the WGS contradicted the finding, and (4) a false negative call, where Gauchian did not report a variant that was expected based on Sanger sequencing. With the total set of true positives and negatives, as well as false positives and negatives, it is then possible to characterize the overall performance using metrics like sensitivity, specificity, accuracy and precision.

Additionally, to further evaluate the frequency of possible false positives, both low (mean depth of 7.4x) and high coverage (mean depth of 47x) WGS data from phase 3 of the 1000 Genomes project^28^ was evaluated by Gauchian. To streamline performance, samples were retrieved in sets of 100 and directly processed in Gauchian utilizing the computational resources of the NIH HPC Biowulf cluster (http://hpc.nih.gov).

## Statistics and reproducibility

Due to the nature of the provided analysis, no statistical analyses were used to explore the data. The data necessary to reproduce the analysis is provided and is used together with Gauchian version 1.0.2.

## Supplementary information

Supplementary Information

Description of Additional Supplementary Files

Supplementary Data 1

Supplementary Data 2.

Supplementary Data 3

Supplementary Data 4

Supplementary Data 5.

Supplementary Data 6.

## Data Availability

The data was submitted to dbGaP, with accession number phs003459.v1.p1.
