# Supplementary material for: Is Gauchian genotyping of GBA1 variants reliable?
Source: Commun Biol. 2025 May 9;8:718. doi: 10.1038/s42003-025-08059-y (PMC12064688; doi:10.1038/s42003-025-08059-y)

Supplementary Figure 1

IGV visualization of whole genome sequencing results for affected exons in patients containing a mismatch between Sanger/WGS validation and the Gauchian prediction.

A – Pat 03

Sanger Annotation: N370S/N370S

Gauchian Call: N370S/WT

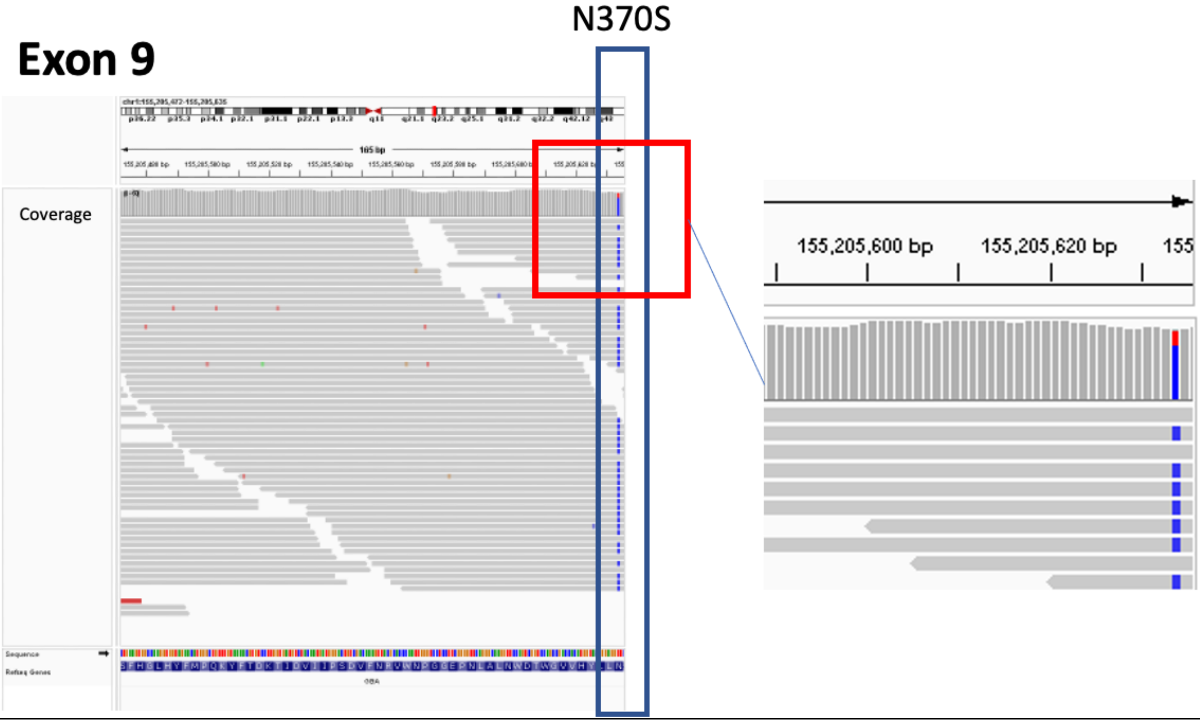

B – Pat_08
Sanger Annotation: N370S/Q350X
Gauchian Call: N370S/WT

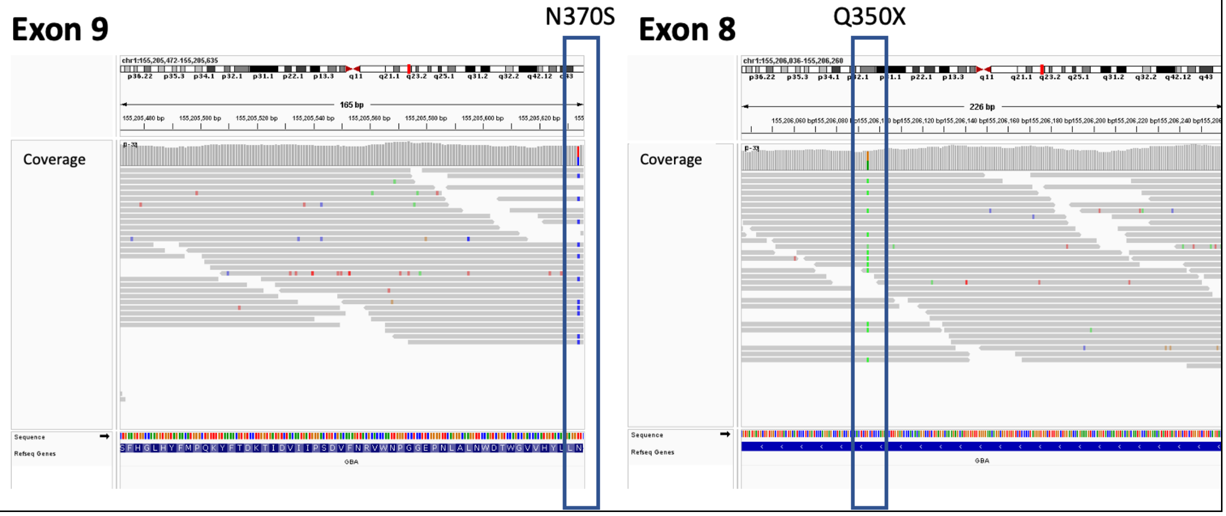

C – Pat_26

Sanger Annotation: N370S/R463H

Gauchian Call: N370S/WT

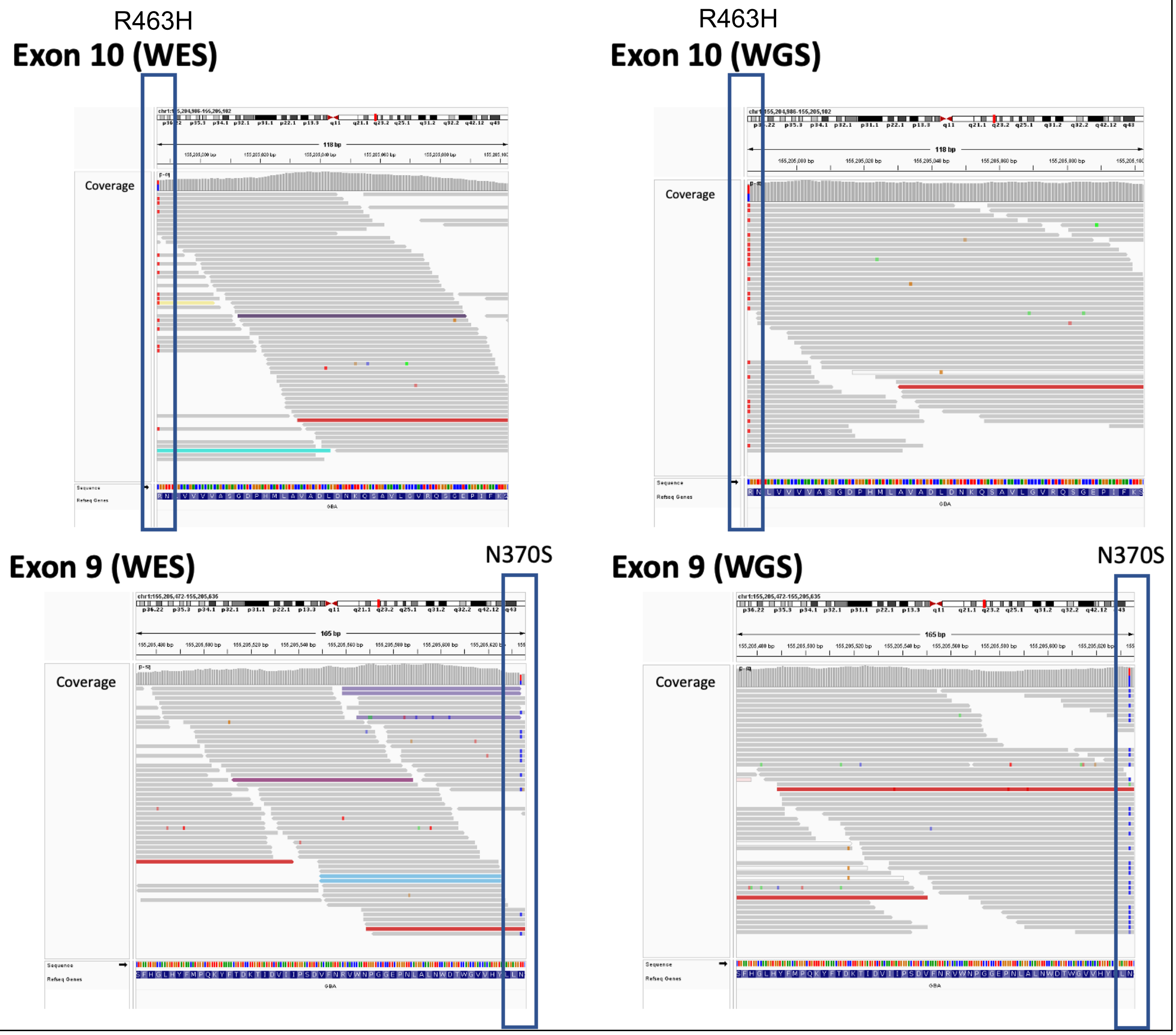

D – Pat_28

Sanger Annotation: R496H/C342Y

Gauchian Call: R496H/WT

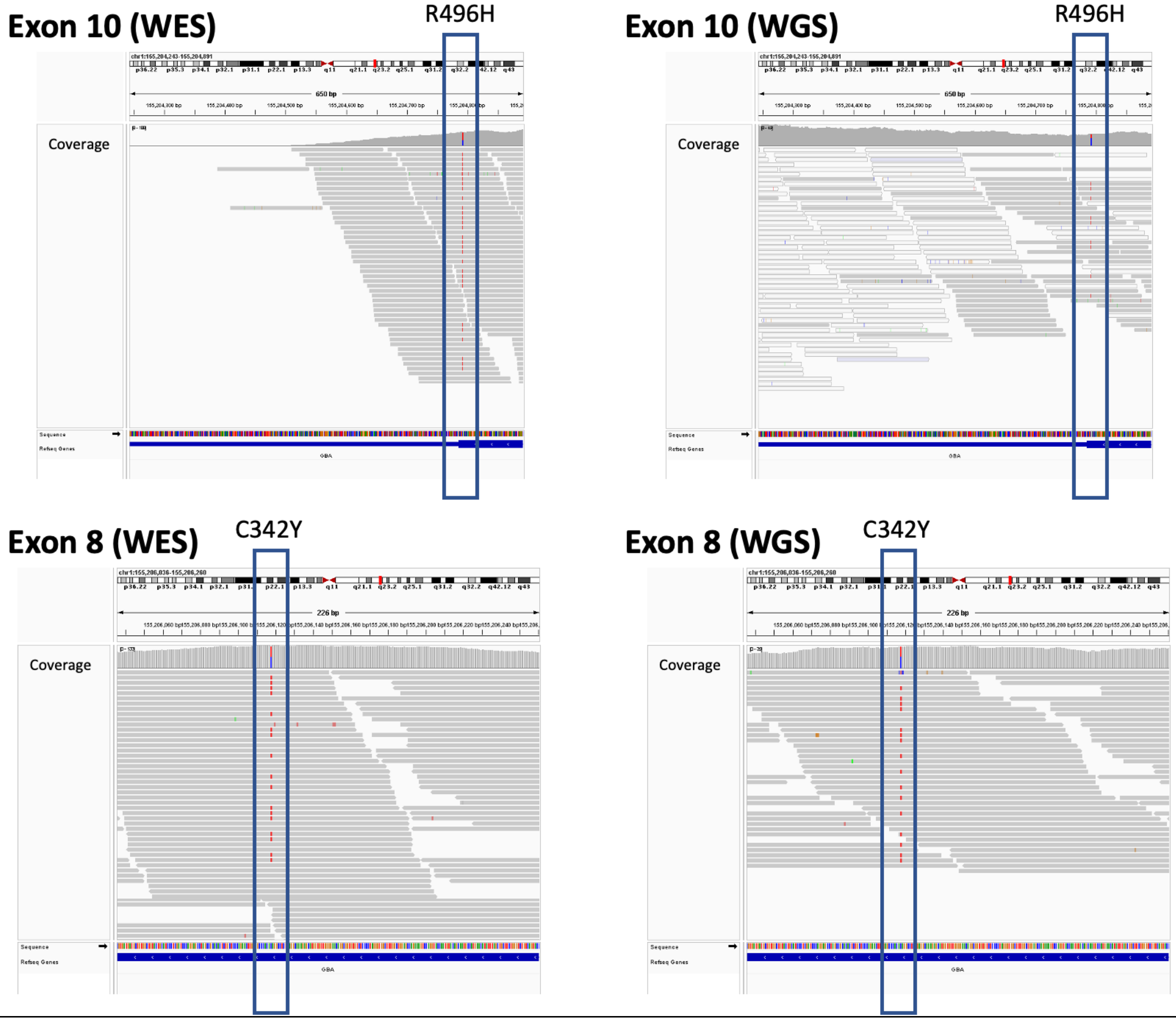

E – Pat_47

Sanger Annotation: N370S/L444P

Gauchian Call: N370S/WT

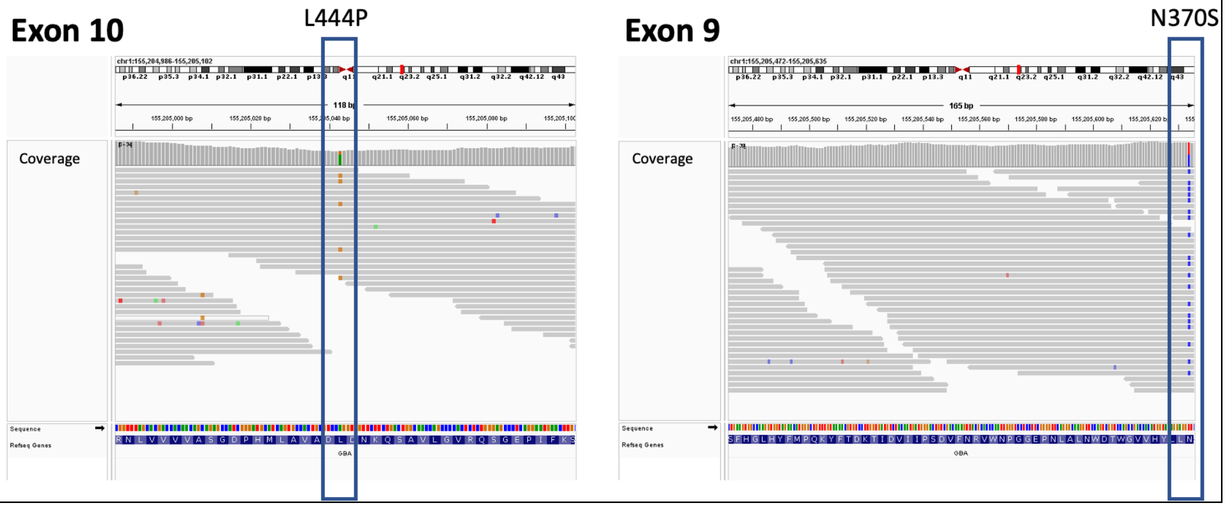

F – Pat_58
Sanger Annotation: N370S/R257*/c203delC
Gauchian Call: N370S/R257*

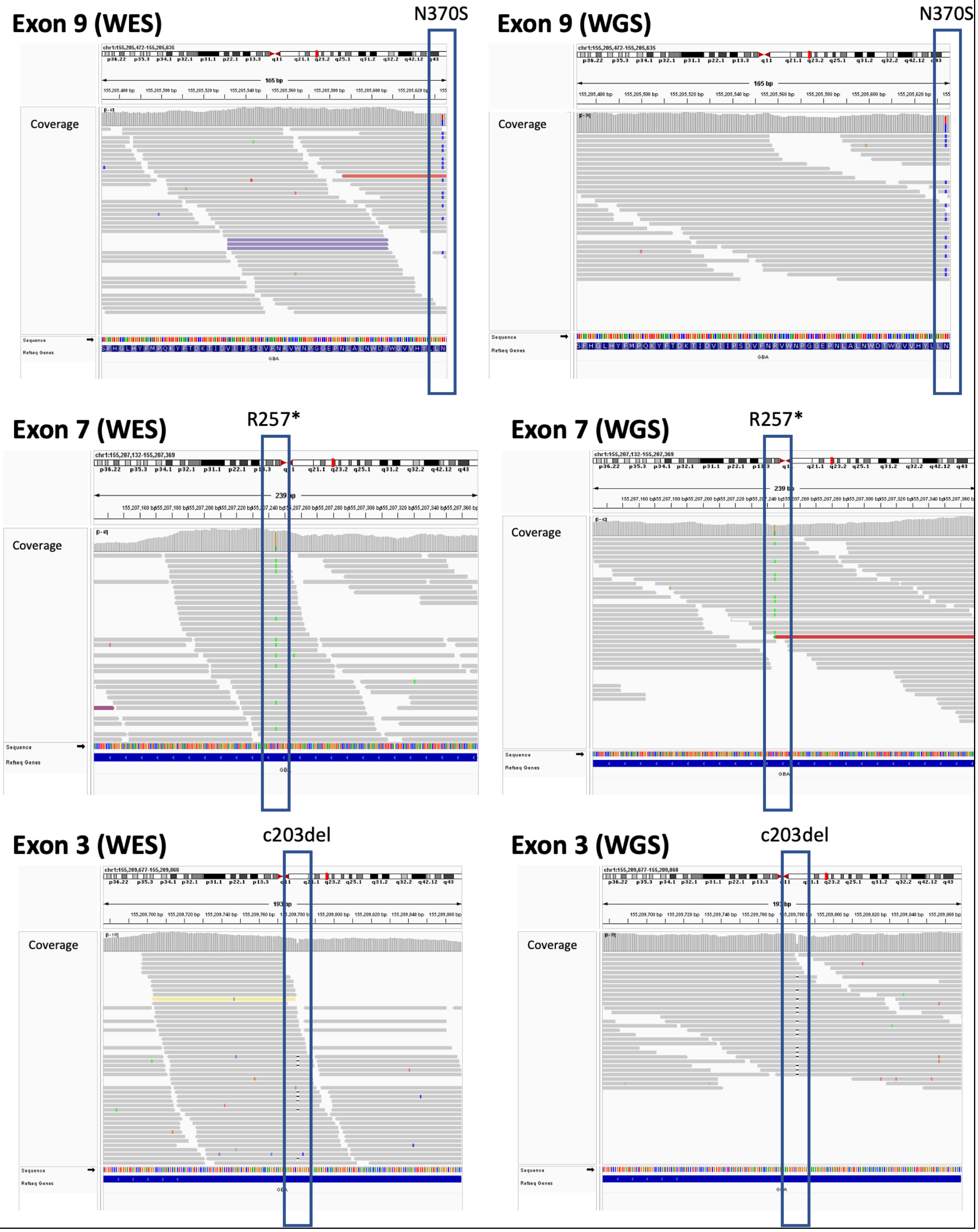

G – Pat_75

Sanger Annotation: R463C/R120W

Gauchian Call: WT/WT

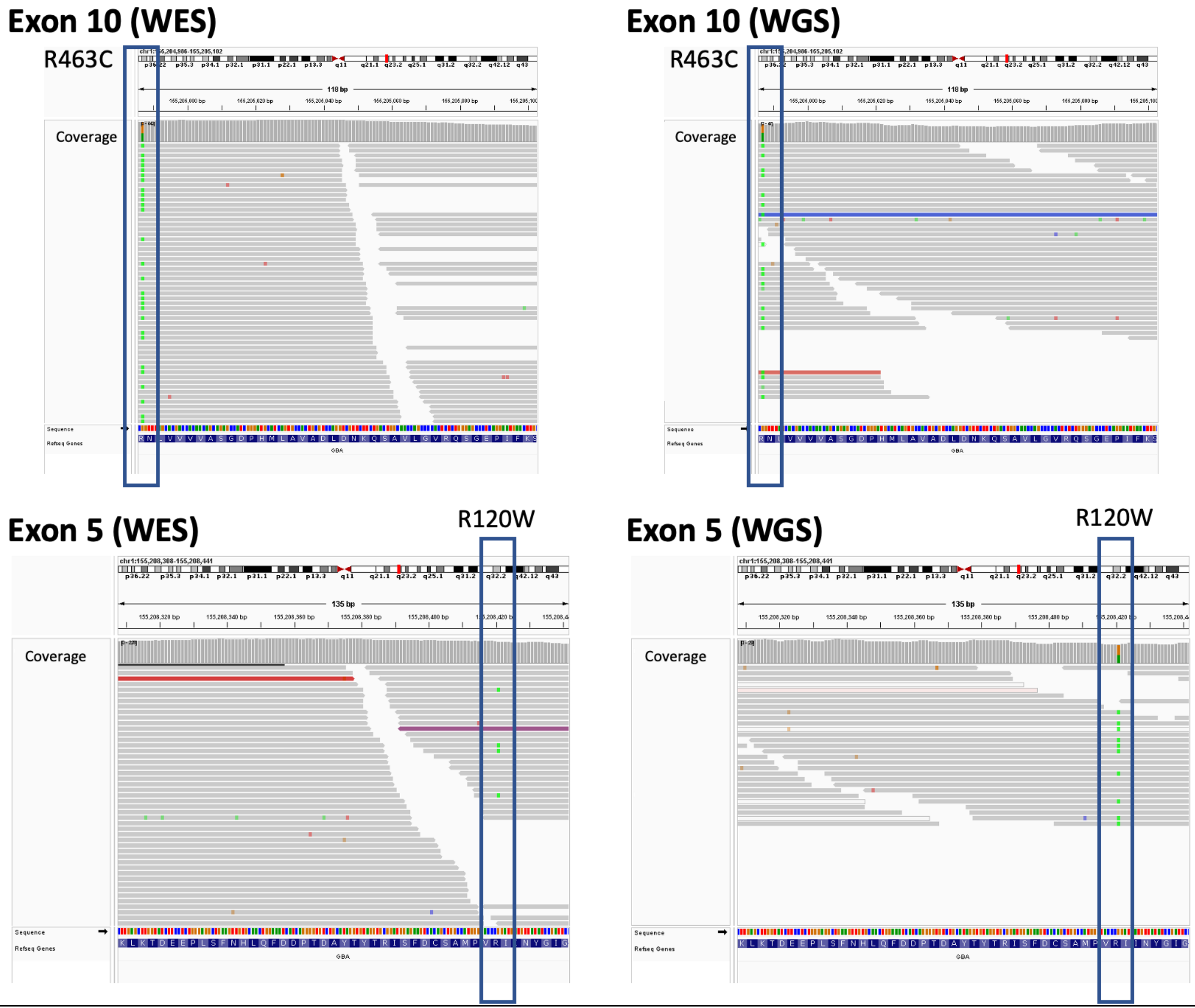

H – Pat_76
Sanger Annotation: N370S/N370S
Gauchian Call: WT/WT

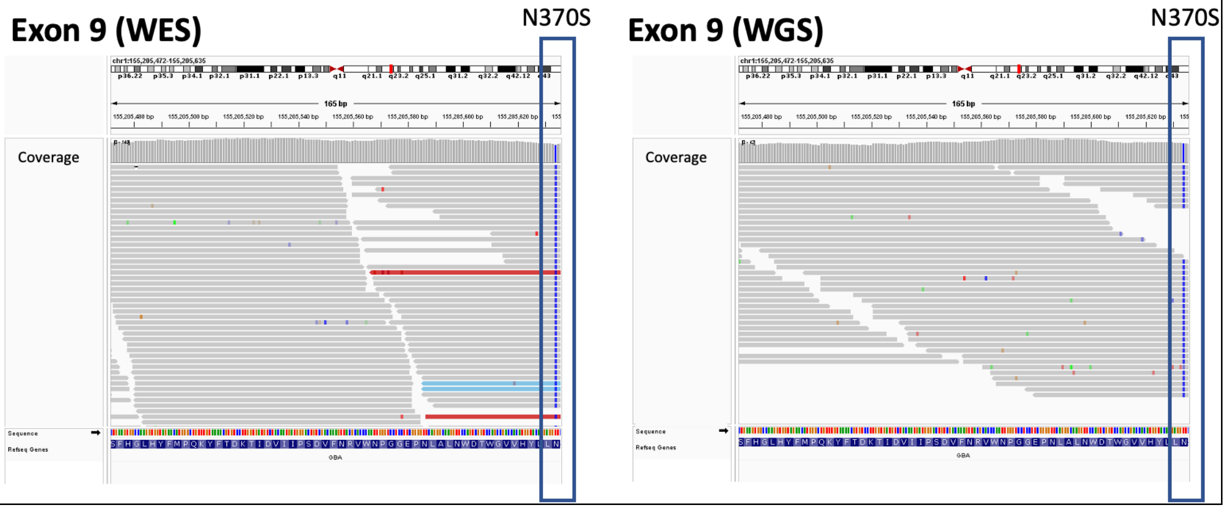

I – Pat_79

Sanger Annotation: L444P/R463C

Gauchian Call: WT/WT

Exon 10 (WGS)

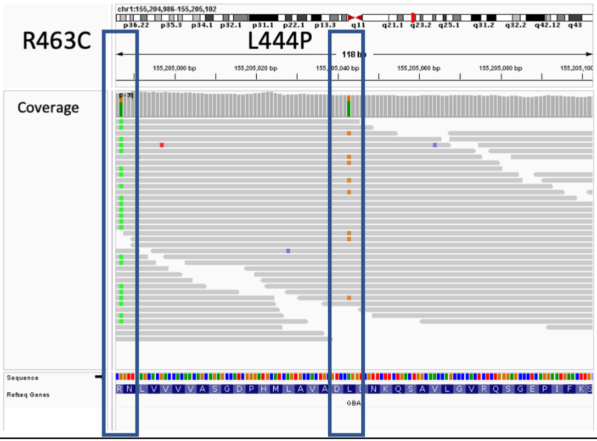

J – Pat_92

Sanger Annotation: D409H/L444P + Rec7

Gauchian Call: D409H/D409H,L444P

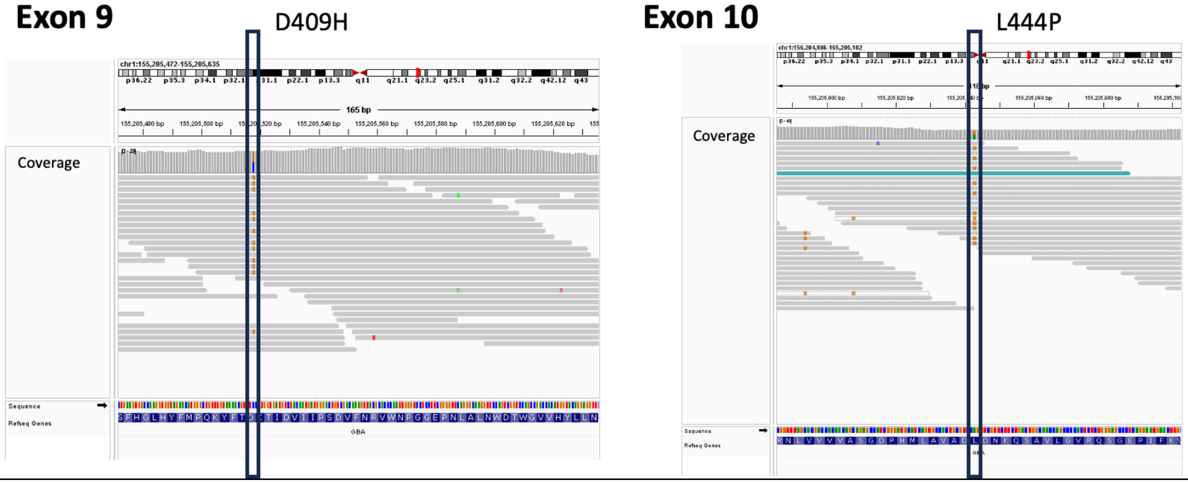

Supplementary Figure 2

Visualization of whole genome sequencing results in patients exhibiting recombinant events.

A – Pat_95 (Rec*Nci*I/p.Asn409Ser)
Exon 9 shows a heterozygous mutation for p.Asn409Ser, while exon 10 shows that the variants associated with RecNciI are not explicitly marked and exon 11 shows that the GBA1/GBAP1 SN mismatch is detectable.

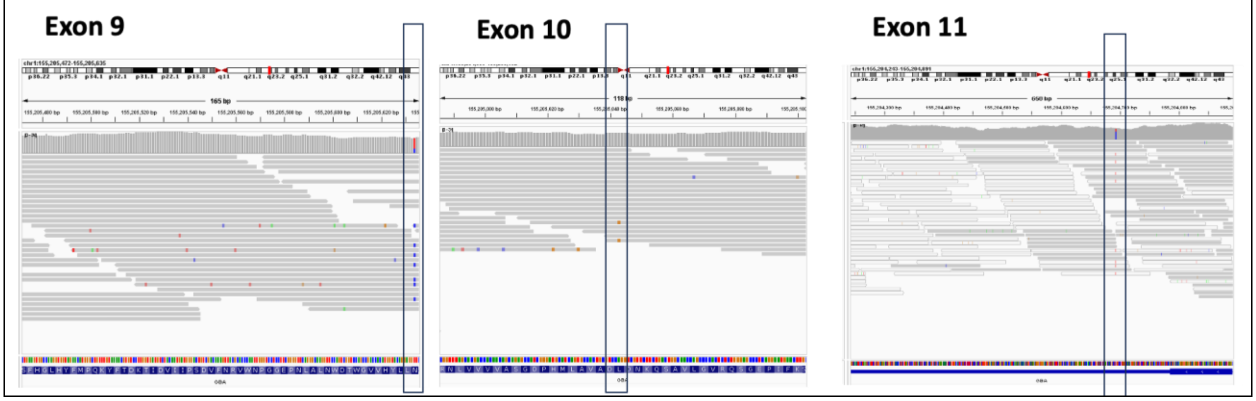

B – Pat_71 (Rec*Nci*I/WT)

Exon 10 shows only p.Leu483Pro detectable for the RecNciI variants and the expected mismatches in the 3’-UTR and Intron 9 are undetectable.

Exon 10

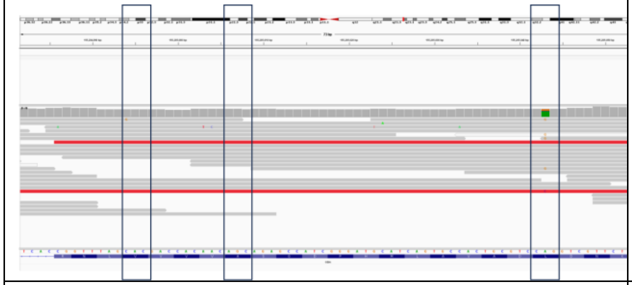

3’UTR

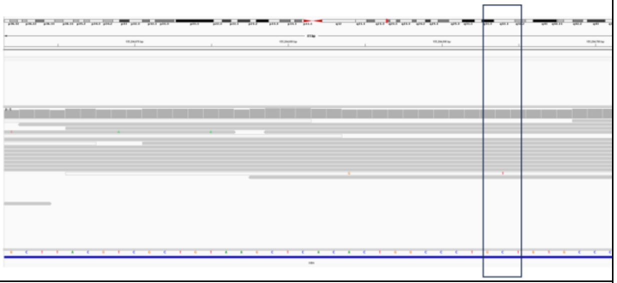

Intron 9

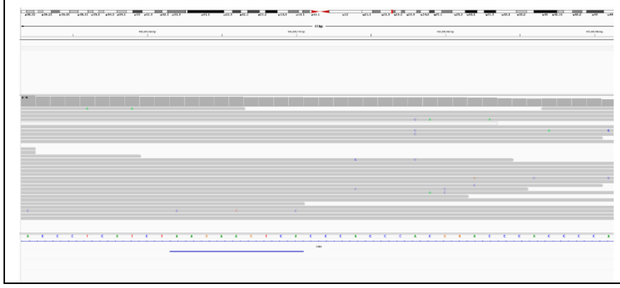

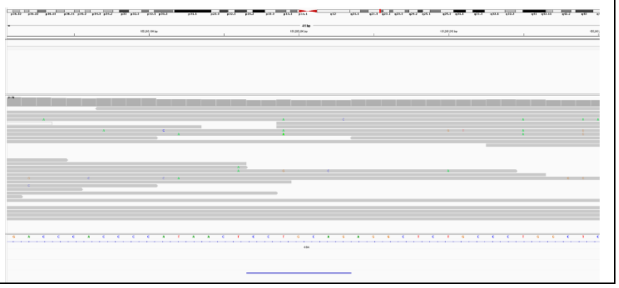

C – Pat_16 (p.Asn409Ser,RecTL+55bpdel)

Exon 9 shows detectable p.Asn409Ser, 55bp deletion but no p.Asp448His. Exon 10 shows that none of the three expected variants are detectable, with the expected mismatches in the 3’UTR also not detectable.

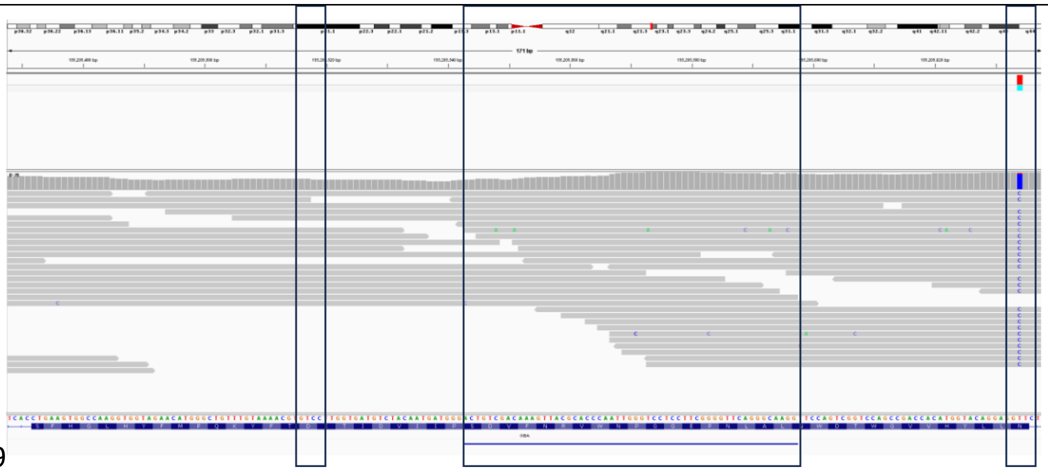

Exon 9

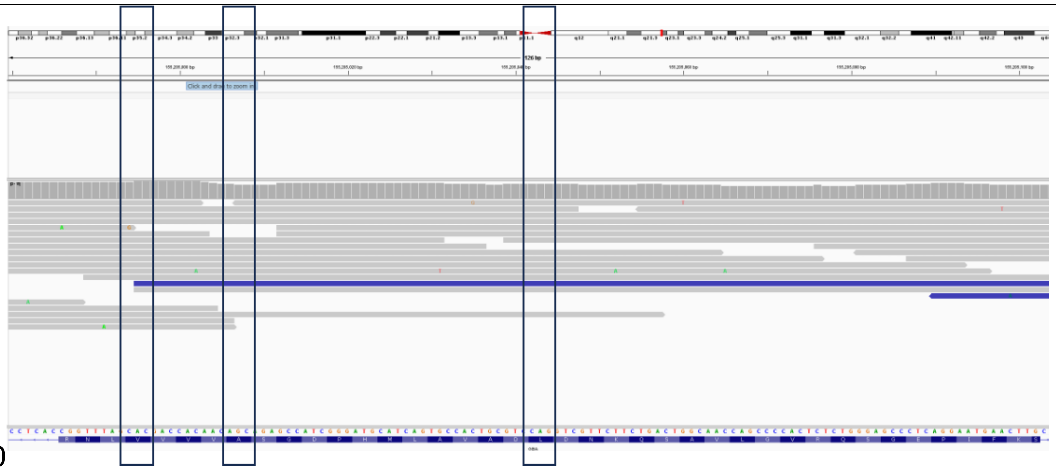

Exon 10

3'UTR

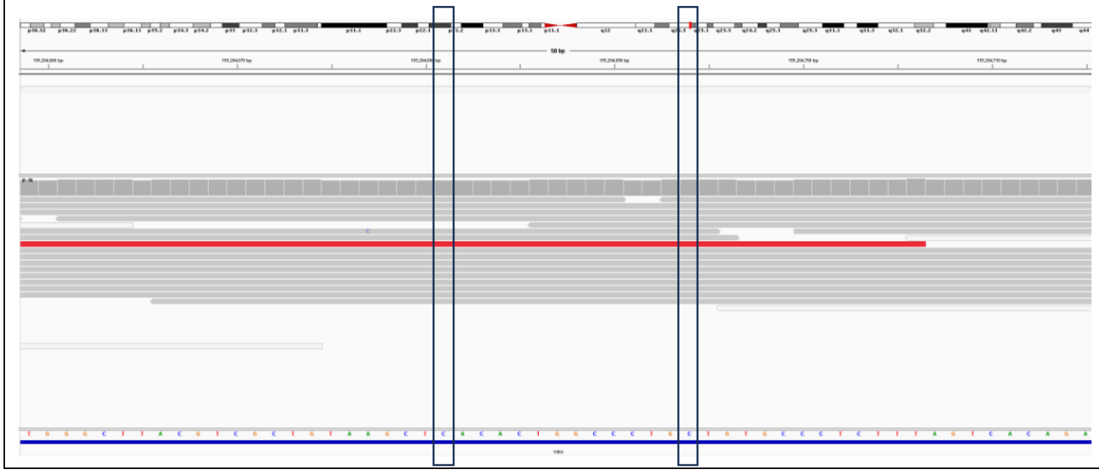

D – Pat_92 (p.Asp448His/p.Leu483Pro, Rec7)
For this *GBA1P* duplication, Gauchian predicted three extra copies (CN=7). The box highlights the possible duplicated area and both missense mutations were detectable.

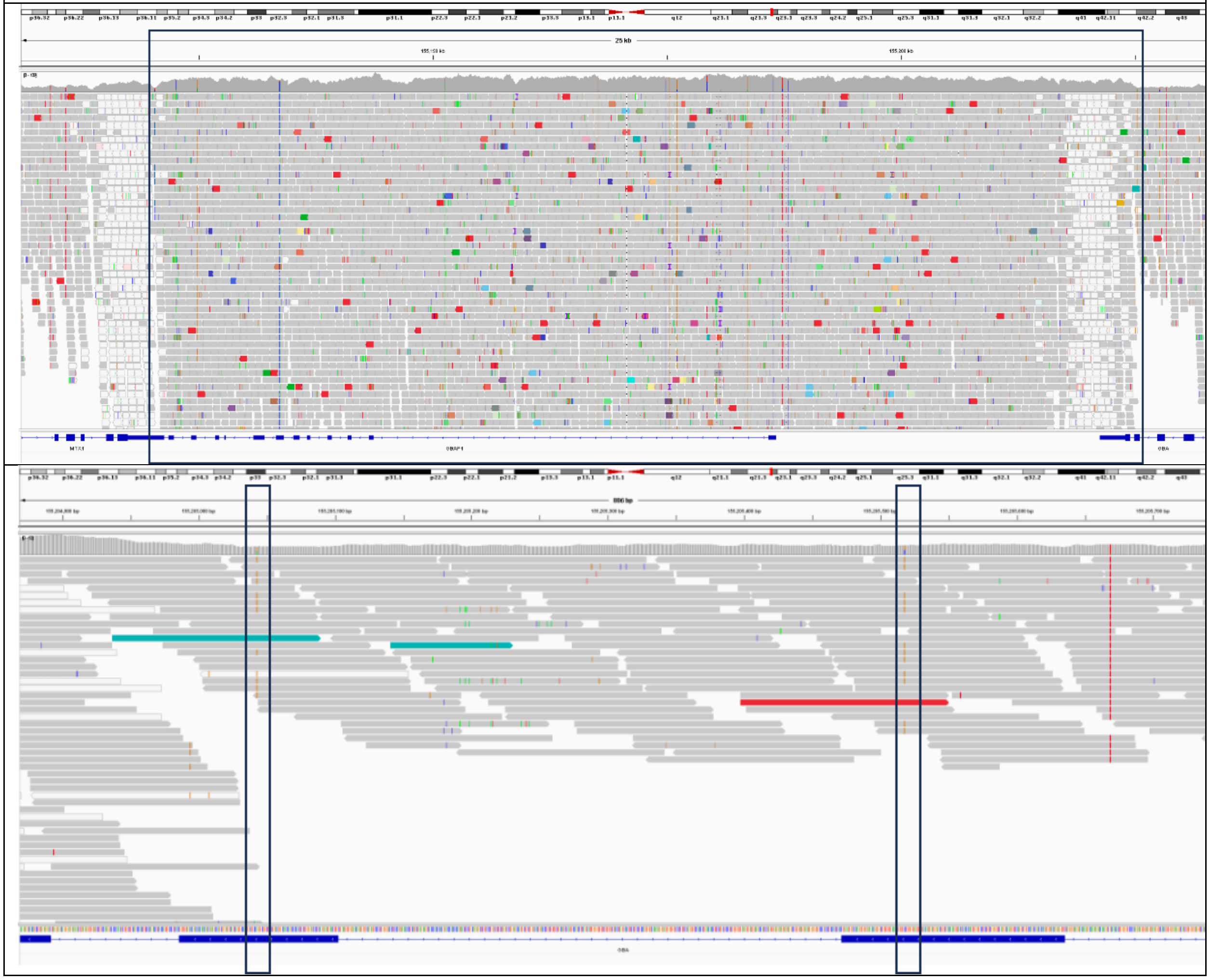

E – Pat _42 (p.Val391Leu/p.Arg398Ter)
For this patient Gauchian predicted two extra copies and a duplication can be detected for *GBAP1*.

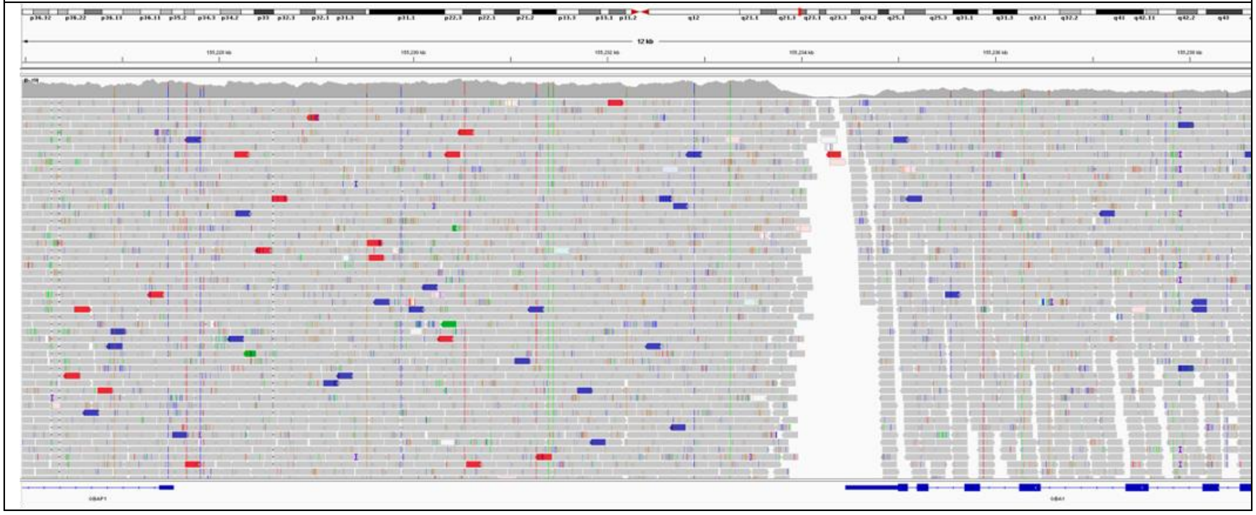

F – Pat_72 (p.Gly241Arg/WT)

For this patient Gauchian predicted one extra copy, which can also be detected for *GBAP1*.

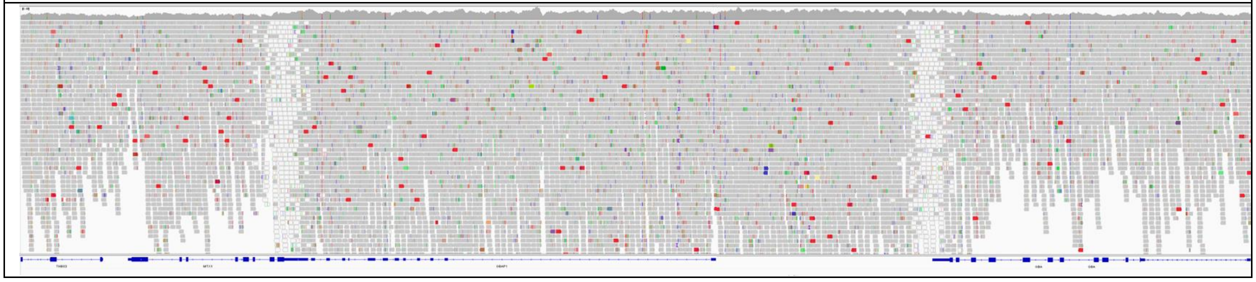

G – Pat_15 (55bpdel/p.Asn409Ser)

The 55bp deletion and the heterozygous p.Asn409Ser mutation in exon 9 are detectable.

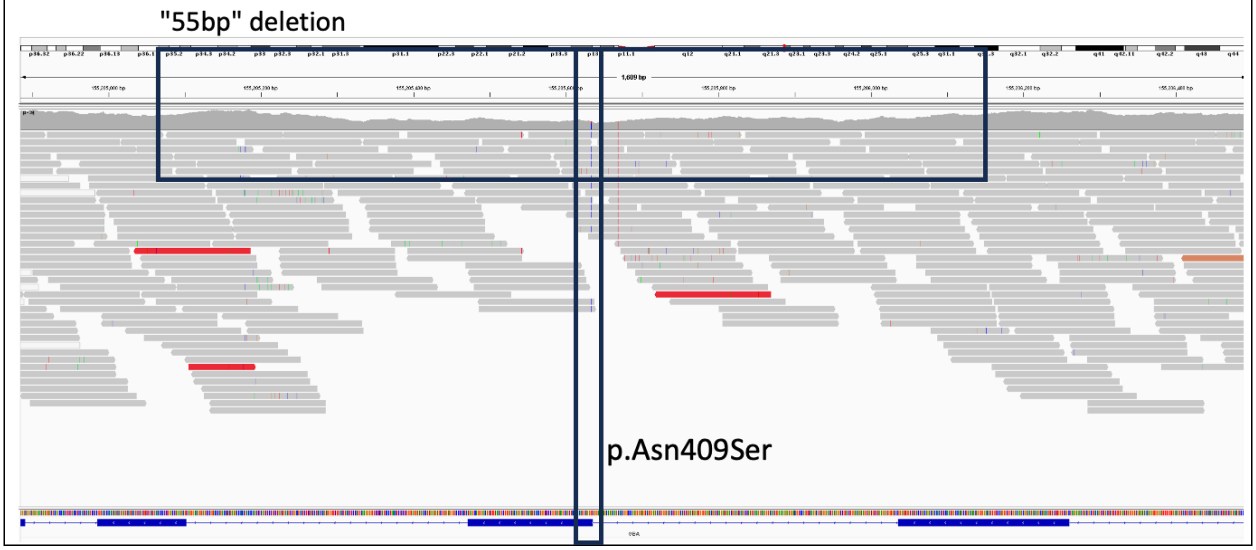

H – Pat_39 (55bpdel/p.Asn409Ser)
The 55bp deletion and the heterozygous p.Asn409Ser mutation in exon 9 are detectable.

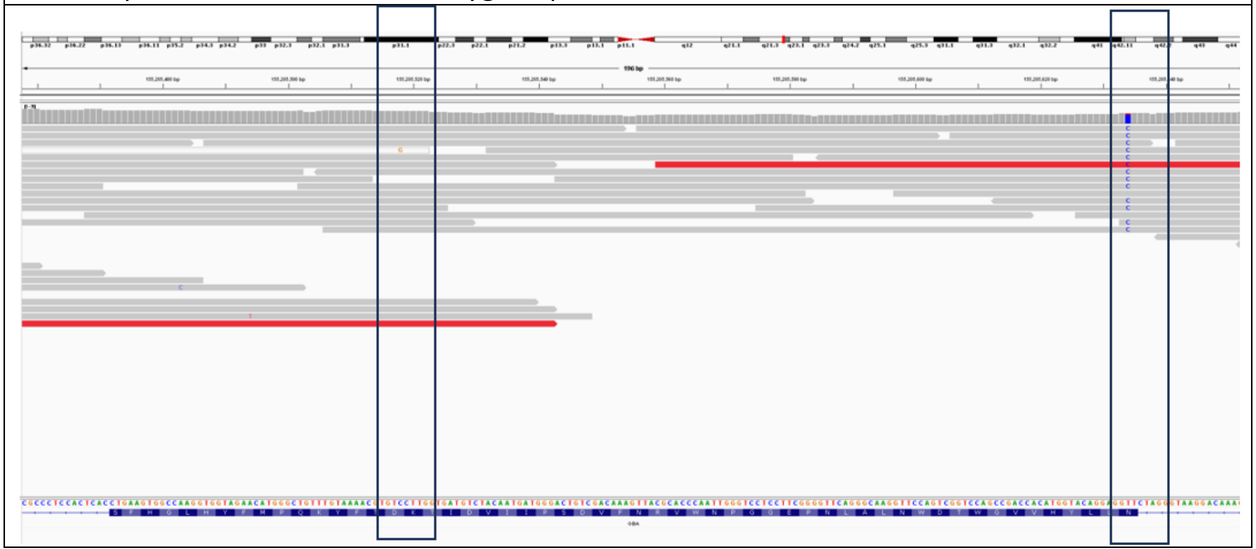

I – Pat_69 (55bpdel/WT)
The 55bp deletion in exon 9 is detectable.

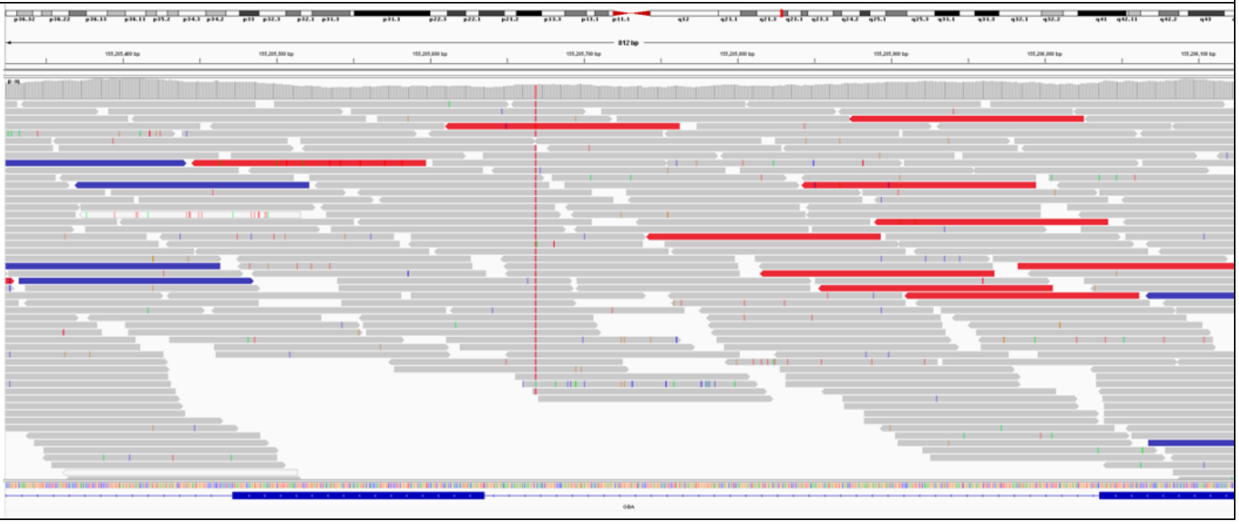

Supplement: Supplementary file 1 — Supplementary Information [file 42003_2025_8059_MOESM1_ESM.pdf]
