# Supplementary material for: Is Gauchian genotyping of GBA1 variants reliable?
Source: Commun Biol. 2025 May 9;8:718. doi: 10.1038/s42003-025-08059-y (PMC12064688; doi:10.1038/s42003-025-08059-y)
Supplement: Supplementary file 2 — Description of Additional Supplementary Files [file 42003_2025_8059_MOESM2_ESM.docx]

# Supplementary Data

Supplementary Data 1. The complete set of 95 whole genome sequencing samples was processed using Gauchian and the results are presented with the original headers provided by the tool. Sanger-established genotype and gender are provided for the NIH cohort and a final assessment comparing the Gauchian call against the Sanger genotype is presented.

Supplementary Data 2. Breakdown of Gauchian performance based on the individual alleles. Sanger sequencing was used for each allele to determine the total number of occurrences of a genotype across all 190 alleles (3 alleles have two variants) as positive identifications and the number of alleles without the genotype as negative identifications. The same approach was used to group the Gauchian predictions into positive and negative calls. Based on these allele-specific groupings, it is then possible to identify true and false positives and true and false negatives. Finally, the sum of incorrectly identified alleles is provided for each genotype and the sums for the different metrics for the complete cohort, corresponding to the entries in a statistical classification-specific confusion matrix.

Supplementary Data 3. Derivations for an allele-specific confusion matrix. Each of the metrics presented is based on ratios of total, true, or false positive and negative counts or other confusion matrix derivations.

Supplementary Data 4. Comparison of Gauchian using b37 and hg38 as reference genomes against the established Sanger calls. Highlighted are rows where the Gauchian calls for b37 and hg38 disagreed.

Supplementary Data 5. The complete set of 95 whole genome sequencing samples from the NIH cohort and low and high-complexity whole genome sequencing samples extracted from the 1000 Genomes project was processed using Gauchian. The results are presented here using the original headers provided by the tool. Each Gauchian genotype was compared against the calls established for the NIH cohort and the 1000 Genomes Project. The comparison of the two genotypes was then broken down into true positive identification and true negative identifications, and each cohort-specific genotype was characterized as a positive call (biallelic *GBA1* mutation), negative call (biallelic wildtype), or a combination of the two.

Supplementary Data 6. Derivations for confusion matrix considering the NIH Cohort, 1000 Genomes Cohort, and the two cohorts combined. Each of the metrics presented is based on ratios of total, accurate, or false positive and negative counts or other confusion matrix derivations.

# Supplementary Figures

Supplementary Figure 1. Visualization of whole genome sequencing results for affected exons in patients containing a mismatch between Sanger/WGS validation and the Gauchian prediction.

Supplementary Figure 2. Visualization of whole genome sequencing results in patients exhibiting recombinant events.
